# Studying the impact of early life exposures to pesticides on the risk of testicular germ cell tumors during adulthood (TESTIS project): study protocol

**DOI:** 10.1186/1471-2407-14-563

**Published:** 2014-08-04

**Authors:** Rémi Béranger, Olivia Pérol, Louis Bujan, Elodie Faure, Jeffrey Blain, Charlotte Le Cornet, Aude Flechon, Barbara Charbotel, Thierry Philip, Joachim Schüz, Béatrice Fervers

**Affiliations:** Unité Cancer et Environnement, Centre Léon Bérard, 28 rue Laennec, 69373 Lyon, 08 Cedex, France; Section of Environment and Radiation, International Agency for Research on Cancer (IARC), Lyon, France; EAM 4128 “Santé Individu Société”, Université Claude Bernard – Lyon 1, Villeurbanne, France; Hôpital Paule de Viguier; Fédération Française des CECOS, CECOS, CHU, Toulouse, France; Université de Toulouse; UPS; Groupe de recherche en Fertilité Humaine (EA 3694, Human Fertility Research Group), Toulouse, France; Centre de Lutte Contre le Cancer, Centre Léon Bérard, Lyon, France; Université de Lyon, F-69003 Lyon, France; Université Lyon 1, UMRESTTE (Unité mixte IFSTTAR/UCBL), Domaine Rockefeller, 69373 Lyon, France

**Keywords:** Case–control studies, Pesticides, Maternal exposure, Paternal exposure, Geographic information systems, Testicular neoplasms, Germinoma, Environmental exposure, Occupational exposures, Gene-environment interaction

## Abstract

**Background:**

The incidence of testicular germ cell tumors (TGCT), the most common cancer in men aged 15 to 45 years, has doubled over the last 30 years in developed countries. Reasons remain unclear but a role of environmental factors, especially during critical periods of development, is strongly suspected. Reliable data on environmental exposure during this critical time period are sparse. Little is known on whether it could be a combined effect of early and later-life exposures.

**Methods/Design:**

Our research aims to study the association between TGCT risk and pesticide exposures (domestic, occupational and environmental) during critical time periods of development and combined early and later-life exposures. The study design, developed during a 2-year pilot study, is a multicenter case–control study of 500 cases (ascertained through histology) and 1000 fertile/fecund controls recruited through 21 French ‘*Centres d’Etude et de Conservation des Œufs et de Sperme humain*’ (CECOS). Trained professional interviewers interview the subjects and their mothers by phone. Using a geographic information system developed and tested for application in this study design, environmental pesticides exposure assessment is based on life-time residential history. Occupational pesticides exposures are assessed by an industrial hygienist based on parents’ occupations and tasks. Exposures during the prenatal period, early childhood and puberty are focused. A blood sample is collected from each participant to assess genetic polymorphisms known to be associated with TGCT risk, as well as to explore gene-environment interactions.

**Discussion:**

The results of our study will contribute to better understanding the causes of TGCT and the rapid increase of its incidence. We explore the effect of combined early and later-life pesticides exposure from multiple sources, as well as potential gene-environment interactions that have until now been rarely studied for TGCT. Our design allows future pooled studies and the bio-bank allows additional genetic or toxicological analyses.

## Background

Testicular Germ Cell Tumors (TGCT, testicular cancer) represent the most frequent cancer in young men aged 15 to 45 years in developed countries with primarily Caucasian populations. TGCT incidence has been increasing throughout Europe over the last 30 years, including in France, where the annual incidence rate has doubled from 3.4/100 000 in 1980 to 7/100 000 in 2008 [1–3]. Large geographical variation in incidence rates exists between different European countries with West–east and North–south gradients [2, 4]. The reasons for such a phenomenon are still unclear but a role of environmental factors is strongly suspected. The rapid increase of TGCT incidence rates and the evolution of the incidence rate in migrant populations [5, 6] support this hypothesis. However, TGCT risk varies also by ethnicity (Caucasian men have a higher TGCT risk than men in Asian or African populations) [7], and familial history of TGCT is also known to be associated with an increased TGCT risk [8], supporting a potential role of genetic factors. It is estimated that 13% of TGCT have a genetic origin [8]. Individual factors have also been suggested to be associated with TGCT risk [9, 10] and several studies have suggested a positive association between a higher socioeconomic status and TGCT occurrence [11–13], although this relationship was not consistently found [14].

Given the peak incidence of TGCT in very young adults and the fact that TGCT has been shown to develop through carcinoma-in situ cells of fetal origin [15], the role of early exposures, in particular during the critical time windows when the reproductive tract develops has been hypothesized [16, 17]. The concept of the Testicular Dysgenesis Syndrome (TDS) proposes that an impaired development of fetal testes may lead to an increased risk of cryptorchidism, hypospadias, testicular cancer and decreased spermatogenesis [17, 18]. However, the TDS incidence in the general population is unknown and to what extent these disorders are actually biologically related through a fetal mechanism remains unresolved. Although the concept of TDS remains controversial [19, 20], the hypothesis of a pre-natal origin of TGCT and a role of in-utero or early childhood exposures to environmental factors in TGCT development remain widely accepted. A combined effect of prenatal, early and later-life (adolescence or adulthood) exposures has also been suggested [21], but has not been explored so far.

It is generally accepted that the development of TGCT is under endocrine control and exposures to chemicals with endocrine disrupting properties, including pesticides, have been suggested to be associated with an increased TGCT risk in epidemiological studies [21, 22]. So far, no appropriate animal models for TGCT exist, therefore our knowledge on factors involved in TGCT development is based on epidemiological research [23]. Reliable data on occupational or environmental risk factors during adulthood are sparse, and only a few studies have investigated parental exposures as a contributing factor to the risk of testicular cancer occurring 20–40 years later [24, 25]. Available studies were often limited by small sample size or by too broad exposure assessment. Furthermore, genetic polymorphisms might be involved in gene-environment interactions by increasing the susceptibility to the effect of endocrine disruptors [26], but these were rarely considered. Additional studies have investigated the place of residence (urban versus rural location), as a surrogate for pesticides exposure, but showed inconsistent results and none of these included the subject’s or parental residential history, or any detailed assessment to environmental pesticides exposure [27–30].

Accurate characterization of environmental pesticides exposure, especially in retrospective studies, is often difficult due to the lack of exposure data available at the individual level, and due to the inherent limitations of conventional epidemiological methods for this type of studies (e.g. recalls bias due to self-reported information). However, some studies have found a positive association between residential proximity to cultivated agricultural fields and pesticides concentrations in biological samples of residents [31–33]. Based on these observations, Geographic information systems (GIS) offer the opportunity to retrospectively assess agricultural pesticides exposures by collecting and analyzing historical environmental data over large areas [34, 35]. To our knowledge, GIS technology has not been applied to study environmental pesticide exposures in relation to TGCT risk.

The discordant findings and the limitations of available studies concerning TGCT risk factors underline the importance to conduct studies with sufficient statistical power to detect risks associated with exposures during critical windows of vulnerability, as well as combined perinatal and later life exposures. The current evidence further underscores the need for studies that can accurately characterize pesticides exposures from multiple sources (environmental, occupational and domestic). Therefore, we conducted a two year pilot study to develop a study design for the case–control study presented here, to compare the effectiveness of different approaches for cases’ , controls’ and mothers’ recruitment in the French context and to verify our ability to collect relevant data on exposures during subjects’ perinatal period (TESTEPERA project) [36]. Based on this pilot study, we chose a national, prospective, face-to-face recruitment. The results also confirmed our capacity to reconstruct cases’ and controls’ occupational and residential history and to accurately geocode most of the addresses (82%), including for early life periods.

### Objectives

Our case–control study aims to assess the impact of multisource pesticides exposure (domestic, occupational and environmental) during prenatal and early childhood periods on the risk to develop a TGCT during adulthood. The study is also designed to assess potential gene-environment interactions as well as the hypotheses of combined prenatal and later life exposure.

## Methods/Design

### Study design

**Figure 1:**
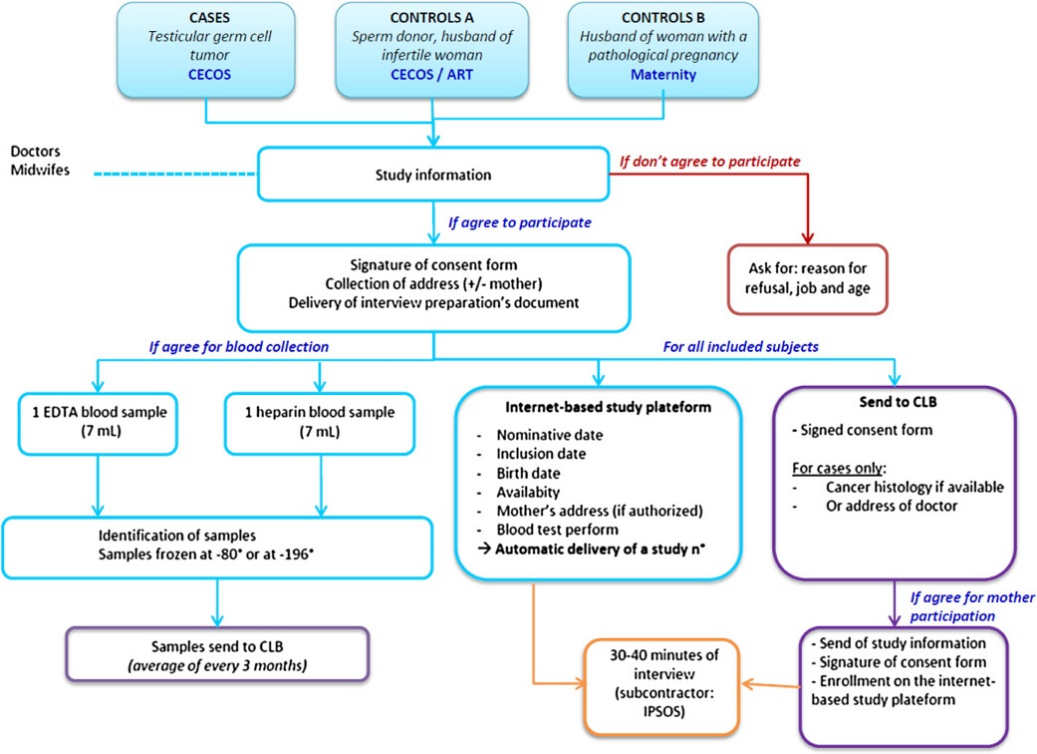
**Schematic organization of the recruitment and the data collection.** ART, Center for Assisted reproductive technology; CECOS, French center for semen conservation; CLB, Centre Léon Bérard; EDTA, Ethylene diamine tetra-acetic acid.

The TESTIS study is a national, multicenter, prospective case–control study of 500 cases and 1000 controls (two groups of 500). Cases are recruited prospectively through the French centers for semen conservation (*Centres d’étude et de conservation d’oeufs et de sperme humain*, CECOS). Controls are recruited in CECOS and centers of assisted reproduction (group A), and regional maternities contiguous to the CECOS centers (group B). Twenty-one out of the 23 French CECOS, included in a national network (*Fédération Française des CECOS*), agreed to participate to the study (Besançon, Bordeaux, Caen, Clermont-Ferrand, Dijon, Grenoble, Lille, Lyon, Marseille, Montpellier, Nancy, Nice, Paris Tenon, Paris Jean Verdier, Paris Cochin, Reims, Rennes, Rouen, Strasbourg, Toulouse, Tours). The study protocol was approved by the French regulatory authorities and by the appropriate French ethics committees (*Comité consultatif sur le traitement de l'information en matière de recherche dans le domaine de la santé (CCTIRS)*; *Comité de protection des personnes (CPP); Agence nationale de sécurité du médicament et des produit de santé (ANSM)*). The study was registered at http://www.clinicaltrials.gov (NCT number: NCT02109926). Figure 1 shows the organization of the study for recruitment, data collection, biological sampling and samples conservation.

### Study population

The study population consists of men aged 18 to 44 years at date of diagnosis, born in metropolitan France, with a valid health insurance affiliation. Cases are men diagnosed with seminoma or non-seminoma TGCT (ascertained through histological report), and seen in one of the participating CECOS for sperm cryopreservation. Controls of group A are sperm donors or partners of infertile women having a normal sperm count (>39 million per ejaculate [37]). Controls of group B are partners of pregnant women hospitalized for a pathological pregnancy in the level III maternity (regional maternity) adjacent to the CECOS. Participants have to sign an informed consent form. Subjects unable to write and understand French language, as well as subjects presenting severe psychological or mental disorders, and subjects under legal guardianship are excluded. Controls with a history of cryptorchidism or of TGCT are also excluded. Two controls (one of each group) are matched to each case on age (±2 years) and the recruiting center.

Considering the incidence of TGCT and the CECOS activity, we estimate a 18-month duration to recruit all cases and controls. The recruitment is performed by physicians and/or midwives (investigators). A written permission is asked to cases and controls to contact their mothers (or the closest relative alive, if the mother is deceased or cannot be interviewed). Participants who sign the informed consent receive a document to prepare information prior to the phone interview, including: lifetime residential history, addresses of schools during childhood, parental occupation at subjects’ conception, birth information, and job history. Investigators propose to each case and control to participate in a blood sample collection. Information on subjects is recorded using a protected on-line study platform that generates a unique identification number for each participant. Cases and controls receive a financial compensation for their participation: 20 euros for completion of the interview and 40 euros for participation in both blood sampling and completion of the interview.

The Principal investigator (PI) contacts case and control mothers (or closest relative alive) upon written permission of cases and controls. Subjects are asked to inform their mother/relative beforehand. Mails include an information letter, a consent form (to be returned using an enclosed pre-paid envelope) and a document to prepare information on residential and job history prior to the interview. Mothers are registered on the same internet platform after the reception of the signed consent form. In absence of any response, up to three phone reminders are done, at two-week intervals.

### Biological sampling and storage

For each case and control that agrees to participate to the blood sampling, two samples are collected at the inclusion by the investigators (1 × 7 ml EDTA (Ethylene diamine tetra-acetic acid) tube, 1 × 7 ml heparinized tube). Blood samples have to be centrifuged within one hour from sampling. For CECOS having -80°C storage, EDTA tube might be stored directly. For CECOS having -196°C storage only (all CECOS are equipped for sperm and egg conservation), buffy coat have to be extracted from the EDTA tube and stored in an adapted cryotube. Concerning the heparinized tube, plasma have to be extracted and stored in 1 ml aliquot in cryotubes at -80°C or -196°C depending on the equipment of each CECOS. Cryotubes are identified using the personal identification number attributed by the online study platform to each participant. Samples are gathered and shipped regularly using a specialized transporter to the Biological Resources Center (BRC) of Centre Léon Bérard (CLB), Lyon. At the BRC, DNA is extracted from the buffy coat (in case of -196°C preservation) or the EDTA tube (in case of -80°C preservation) using the AUTOPURE automaton (Quiagen, Germany). Then, DNA is stored at -80°C (300 ng DNA per aliquot).

### Data collection

Data from cases, controls and case/control mothers (or relative) are collected through a standardized phone questionnaire administrated by professional interviewers (IPSOS company). Interviewers are unaware of the case or control status of study subjects. To ensure consistency in data collection, interviewers have been trained in the completion of the questionnaire and provided with a field guide. All data are entered directly on the specially designed online study platform used for registration and data collection (for items, see Table 1). Investigators, technicians and researchers have a personal login and password to access the platform. Accesses to sensitive data are restricted, based on the user profile. The coordinating center contact the clinicians in charge of cases to obtain the pathology report and serum markers (alpha feto-protein, beta-HCG). For eligible subjects who refuse to participate, age, job, and reason for refusal are collected by investigators and entered into the study platform.

**Table 1:** **Items collected during the phone interview**

Categories	Cases and controls	Mothers (or closest relatives)
General information	Medical history and long term treatments (childhood); Birth characteristics; Geographical origin; Socio-economic status	Medical history (mother); Treatments during pregnancy; Age and morphology at birth; Birth characteristics (son); Socio-economic status
Occupational exposures	Entire job history (+tasks and company name and addresses); Specific questions on pesticides, solvents, welding fumes, heavy metals and plastic exposures	Job history from the beginning to the 17 years of the son (mother)/job history from 1 year before the conception to the 17 years of the son (father); Specific questions on pesticides, solvents, welding fumes, heavy metals and plastic exposures
Environmental exposures	Whole residential history and households characteristics; Addresses of schools	Residential history from 1 year before son’s conception to the 17 years of the son
Domestic exposures	Domestic use of pesticides gardening, pet treatment, indoor usage of insecticides or fungicides, and lice treatment (at puberty)	Domestic use of pesticides gardening, pet treatment, indoor usage of insecticides or fungicides, and lice treatment (son: perinatal period and at puberty)
Lifestyle	Smoking status; Drug use; Physical activity	Smoking status; Drug use

### Occupational exposure assessment

Occupational exposure assessment of prenatal and early postnatal periods involve encoding parental occupations (mother: from the beginning of the job history to the 17 years old of the subject; father: from one year before conception to the 17 years of the subject). Based on the occupational history and job/task related information, all occupations are encoded by an industrial hygienist according to the International Standard Classification of Occupations (ISCO). The ISCO-68 is used to classify jobs of subject’s parents, and the ISCO-08 is used for the subject’s jobs. The French nomenclature of activity (NAF) is used to code the industrial classification of all jobs. In a second step, an industrial hygienist performs a detailed occupational exposure assessment based on job and task descriptions. Specific items have been added to the questionnaire to help the hygienist to assess exposures suspected to be associated with TGCT: pesticides, plasticizers, solvents, welding fumes and heavy metals. For each job held, probability, intensity (low, intermediate, strong) and duration of exposure are encoded.

### Domestic exposure assessment

Domestic exposure to pesticides is assessed for cases, controls and their mothers. Specific items in the questionnaire cover the main domestic pesticide use by interviewees and persons sharing the same household (gardening, pet treatment, indoor usage of insecticides or fungicides, and lice treatment), as well as the frequency of use. Pesticide exposure (compound family, probability of exposure and intensity) are estimated through expert assessment, based on the pesticide-use matrix developed by the National Cancer Institute (MD, US) [38].

### Environmental exposure assessment

Cases and controls residential history are gathered from 1 year prior to birth to date of the inclusion in the study. Semi-automatized fields in the study platform help to reduce misspellings when entering the questionnaire data. In case of inconsistency between subjects’ and mothers’ information, data provided by the mothers are used. Addresses are geocoded using the database “*BD adresse*” from the French National Geographic Institute (IGN), which contains coordinates of all addresses in France (unit: RGF Lambert 93). Since the coordinates of the database are centered on the postal address, a GIS technician moves manually the point to the center of the household. Using dedicated software (BD Adresse® for ArcGIS Locator), we identify all addresses geocoded with poor precision (at the street level or less) for manual verification or repositioning (when possible). Specific additional questions are added to the questionnaire to help the GIS technician when no street number is available (closest crossing road or point of interest).

The use of infra-red images, based on the greenness reflectance, has been used successfully in previous US studies to reconstruct land use data [39, 40]. Satellites images are available from the Landsat® program from 1972 with an 80 m spatial resolution and from the SPOT® program from 1986 with a 20 m spatial resolution. Based on remote sensing of satellite images and/or photo interpretation of IGN aerial photography (available from 1920’s), we determine the land use around each residence of interest. When data are not available or of poor quality, we assign land use data of the nearest available time period to the residence. Public data from agricultural statistics (Recensement Statistique Agricole, DRAAF) as well as expert assessment are used to validate our land use layer, when needed. The agricultural statistics provide the proportion of each type of crops in each municipality for 1970, 1979, 1988, 2000, and 2010.

The GIS based approach has been developed in a previous study (SIGEXPO project [41]). We investigated the link between environmental parameters (crop acreage, characteristics of neighboring cultivated fields, geographic and meteorological variables) and the concentration of pesticides in indoor dust of nearby homes. More than 700 samples were collected from 239 volunteer homes in the Rhône-Alpes region, France. These were distributed according to the different types of territories and according to different levels of intensity of theoretical exposure. Samples were taken during the main period of pesticides use according to the representative cultures of the Rhône-Alpes region (orchards, wine, cereals), reflecting the main application modes used in France (rotary atomizer, inflatable ramps, pneumatic sprayer, and motorized mist blower mounted on straddle tractors). According to this study, our GIS methodology is based on 500 meter and 1000 meters buffers, the frequency of the wind direction (data from Meteo France®), the presence of topographic barriers (BD Alti, IGN), vegetative barriers (BD Topo®, aerial photography and/or remote sensing), and structural barriers (BD Topo®, aerial photography and/or remote sensing). The score we developed (Agricultural Exposure Index (AEI)) estimates the intensity of exposure to the different crop types for each address/year.

Specific attention is given to locations during known or suspected critical lifetime periods in the etiology of TGCT (prenatal, early life, puberty). The AEI can be used as a surrogate of agricultural pesticide exposure level. In a second step, we use pesticides matrices to convert the crop exposure level into a pesticide exposure level, for each family of pesticides (or compound per compound, when available). These matrices contain the list of pesticides likely to have been used depending on the type of crop and period. Two matrices are currently under construction in France and might be used in our study: MATPHYTO (*Institut de Veille Sanitaire, France*) and PESTIMAT (*Institut de Santé Publique, d'Épidémiologie et de Développement, France*).

### Social deprivation and territorial indicators

To determine the impact of social deprivation on TGCT risk, using the Townsend index [42] and European Deprivation Index (EDI) [43], individual and territorial socio-economic data are collected to be included in the GIS. Territorial data are available from INSEE (French National Institute for Statistics and Economic Studies) at the IRIS scale (acronym for ‘aggregated units for statistical information’, covering a target size of 2000 residents per basic unit).

### Genetic analyses

We investigate polymorphisms known to be associated with TGCT risk. We identified 45 Single Nucleotide Polymorphisms (SNPs) revealed through 4 Genome Wide Association Studies and one replication study (from 8 loci: KITL, BAK1, SPRY4, ATF7IP, TERT, DMRT1, TGFBR3 and BMP7) [8, 44–47]. Associated odds ratios (OR) were 1.37 (95%IC 1.1 – 1.58, p = 10 e-13) for polymorphisms on chromosome 5; 1.50 (95%IC 1.28 – 1.75, p = 10 e-13) for polymorphisms on chromosome 6; and 2.55 (95%IC 2.05-3.19, p = 10 e-31) for polymorphisms on chromosome 12. Additionally, we identified 6 additional SNPs associated with organochlorine metabolism pathways and known to be able to modify the risk to develop a TGCT (2 loci: CYP1A1 and HSD17B4) [26]. Other polymorphisms may be added if new publications suggest additional polymorphisms prior to the genetic analysis. Considering rapidly decreasing costs of genetic analyses, a genome wide screening might become an alternative option for SNPs analyses.

### Determination of the sample size

Our research will examine the association between TGCT risk and occupational, domestic and environmental pesticides exposure. Since occupational pesticides exposure is supposed to be less frequent and associated with higher exposure levels than domestic and environmental exposures, we based our sample size calculation on the prevalence of occupational pesticides exposure among case and control parents. Based on the prevalence of agricultural workers in France in 1988 (10%) (http://agreste.agriculture.gouv.fr/IMG/pdf/AGRIFRA07c-2.pdf), the minimum detectable odds ratio with our sample (500 cases and 1000 matched controls) is 1.6, considering a statistical power of 80% at a significance level of 5%. Considering additional occupations associated with pesticides exposure (e.g.: greenhouse worker, sawmill worker, forester), the total prevalence of exposed workers should be higher. Considering a prevalence of exposure of 15%, the minimum detectable odds ratio under the conditions outlined above is 1.5.

### Statistical analyses

Standard descriptive statistics will be used to describe characteristics and pesticide exposure of cases, controls and mothers/relatives. Exposure variables will be explored using supervised principal component analyses [48]. Risk analyses will be based on conditional logistic regression models, to compute odds ratios for TGCT at different levels of exposure. Exposure variables will be investigated as continuous variables (with appropriate transformation to achieve normality) as well as categorical variables (quartiles or predefined categories depending on variable type). In addition we will create a combined pesticide exposure variable based on pesticide exposure (occupational, environmental and domestic) during prenatal and early postnatal period (PEPPP) and during adolescence (PEA). We will examine PEPPP exposure, PEA exposure, and combined PEPPP and PEA exposure in relation to TGCT risk. The effects of additional potential confounders (other than our matching criteria) on the associations between pesticide exposure and risk of TGCT will be examined and added to the model one by one. Comparison between models with and without adjustment will be used to examine the potential confounding effect of these factors and only factors with relevant changes in the odds ratios will be kept in the final model. Potential confounders include the geographical origin, socio-economic status, tobacco and cannabis consumption, length and weight at birth, birth order, and the familial history of TGCT.

The two control groups will be used first as separate control groups to identify potential major differences. In order to examine whether any of the associations between pesticide exposures and TGCT risk differ by subgroups of known risk factors or by genetic polymorphisms, we will perform additional stratified analyses. Tests for statistical interaction will be used to examine whether any apparent heterogeneity of effect is statistically significant. This will be done by comparing models with and without interaction terms between the risk factor or genetic polymorphisms and the environmental variable (pesticide exposure) with a maximum likelihood ratio test. Sensitivity analyses are planned to explore the impact of potential bias or methodology limitations (such as quality of satellite images, through removing subject born before 1986 having less precise satellite images at time of birth).

### Steering committee

A Steering Committee has been implemented to oversee the study progress. It is composed of the two principal investigators, a project manager, a doctoral student, study partners, two oncologists specialized in TGCT and eight CECOS representatives. The Steering Committee will meet every 4 months. It will be regularly informed about study progress and of any emerging problems. It will monitor compliance with the study protocol, the quality of collected data and will review scientific reports and publications.

## Discussion

Considering the rarity and latency of TGCT, the case–control design appears to be the most appropriate method for our research. Based on a two-year pilot study, the TESTIS project was optimized to address short comings identified by previous research despite the rarity of the TGCT. In France, around 2000 men are diagnosed with TGCT each year and at least 1100 TGCT patients are seen annually for sperm cryopreservation in the CECOS network. The CECOS have a regional recruitment, and only the CECOS are allowed to cryopreserve sperm in France. Based on our pilot study, loss due to inclusion/exclusion criteria and to non-participation should not exceed 50%. Thus, we estimate that 18 months is long enough to ensure the recruitment of 500 TGCT cases in the 21 participating CECOS.

Testicular cancer and reproduction are sensitive topics. Recruiting the controls through hospital settings aims to facilitate the recruitment of our young male population, as well as managing biological sampling. To ensure that both cases and controls are recruited at a regional level, we decided to select Group B controls among partners of women having a pathological pregnancy. The latter are managed centrally in the regional maternities adjacent to the participating CECOS, while selecting controls among partners of all pregnant women in these maternities would lead to over-representation of the urban population. With a participation of 21 out of 23 CECOS, we assume that our sample is representative to the French metropolitan territory.

In general, young men are difficult to approach and less likely to participate to research than other population groups. Low response rates make difficult ascertaining a population-based control group representative of the general population. Since no perfect control group was found, we choose two distinct control groups to test our hypotheses on populations presenting different aspects of the general population, as made by Stang et al. [49]. Controls from Group A and Group B present both advantages and weaknesses (see Table 2). Associations found consistently in the two control groups separately will strengthen our hypotheses, whereas inconsistent findings will provide new insights on potential confounding factors.

**Table 2:** **Main advantages and weaknesses of the two control groups**

	Advantages	Weaknesses
Control Group A *(sperm donators & fertile partners of infertile woman)*	- Direct access to the subject (face to face recruitment & blood sampling)	- Older than cases/difficult to recruit subjects below 25 years old
	- More concerned by the topic/good participation rate	- Live with infertile woman/more exposed to reproductive toxicant than general population?
	- Sperm count available	
	- Regional recruitment	
Control Group B *(partners of pregnant woman hospitalized for pathological pregnancy)*	- Same age group than cases	- More difficult to approach (visit during evening/week-end)
	- Direct access to the subject (face to face recruitment & blood sampling)	
	- Presumably fecund	- No available serologies (need to store blood samples in separate areas)
	- Regional recruitment	- Link between subjects’ exposures partners’ pathological pregnancy?
	- Large population/easy to match with cases	

According to the TDS hypotheses, TGCT, cryptorchidism, hypospadias and several forms of male infertility are suspected to share common etiological factors [17, 18]. By choosing controls supposed to be fecund or having a normal sperm count, we aim to avoid subjects suffering from a minor form of TDS. In consequence, our controls are likely to be more fertile than the general population of the same age, and may present lower exposure to reproductive toxicants during postnatal periods. This should be taken into account in the interpretation of results when considering adolescent or adulthood exposure. Also, setting up a third control group, more representative of the general population (e.g. by selecting healthy young males from an existing cohort study conducted in France), may be considered.

The financial compensation for cases and controls participation will help recruitment, although the amount of compensation is kept low so subjects are not tempted to participate in the research against their personal convictions. Minimal information (age, occupation and reason for refusal) will be collected from subjects refusing participation to compare participants and non-participants and identify differential non-participation. Additionally, our study population may be compared to the general population based on data from the French National Institute for Statistic and Economic Studies (INSEE). This step will require coding all jobs according to the French classification of jobs and socio-economic categories from 2003 (PCS-2003).

Since prenatal information is of major interest to our research, we decided to include mothers of cases and controls in our study. Previous studies shown that mothers’ participation rate range from 54% to 71% [50, 51], which was similar to our pilot study [36]. Minimum information regarding parental occupation and residential addresses during the prenatal period is included in the case and control questionnaire to reduce missing data in case of mother’s non participation. Reliability of these items will be assessed by comparing information collected from participating case and control mothers and their respective sons.

Our biological sample collection will allow further ancillary projects. Heparinized plasma may be used to search for organic pollutants (multiresidue analyses) or biomarkers, whereas DNA samples will allow participating in genome wide association studies or perform DNA methylation studies. Biological samples will be made at the time of diagnosis, before any radio- or chemo-therapy (information recorded at time of the inclusion).

To reduce recall bias and to minimize missing data, we use objective criteria for exposure assessment when possible (job history analyzed by an industrial hygienist; use of GIS methods based on residential history). All subjects receive a document to gather information on residential and job history prior to the interview. The use of trained interviewers, blinded to the case or control status will ensure the same degree of questioning for both cases and controls.

A risk of misclassification for the GIS method remains due to imprecision in geocoding of residential addresses. Our pilot study allowed precise geocoding for 82% of all subjects’ residences. Lower precision was significantly associated with size of communes (<10,000 inhabitant), mainly related to rural addresses or hamlet lacking street numbers. No statistical difference in accuracy was found according to time period. In a recent French study, Semalgue-Faure et al. estimated that the medium imprecision of addresses when placed at the center of the hamlet or the street was about 200 meters [52]. Our geocoding accuracy was similar to this study [52], but slightly lower compared to several US studies [53–55]. However, geocoding of US addresses showed decreasing precision for older addresses [53]. Yet, imprecision does not lead to differential misclassifications. To improve ascertainment of the most accurate addresses, instructions have been given to interviewers on how to obtain complete addresses, or if not available, request ancillary information such as names of nearest intersecting roads or a nearby landmark still likely to be in place.

We expect our results to contribute to better understanding of the causes of TGCT and the rapid increase of its incidence. Thanks to the interdisciplinary network of research teams, we will be able to explore the effect of combined early and later-life pesticides exposure from multiple sources, as well as potential gene-environment interaction that have been poorly studied for TGCT. The use of GIS to assess environmental exposure to agricultural pesticide as well as the combination of environmental, domestic and occupational exposure are innovative and will improve exposure characterization. The thorough geocoding of subjects’ lifetime residential history will allow analyses of additional environmental risk factors in future studies as new hypothesis emerge. This research is an innovative approach in France that will contribute to improve our knowledge on the long term effects of pesticide exposure on human development, and potentially provide support for decisions in future healthcare policies.
